# Preparation of V_2_O_5_ Composite Cathode Material Based on In Situ Intercalated Polyaniline and Its High-Performance Aqueous Zinc-Ion Battery Applications

**DOI:** 10.3390/ma18102166

**Published:** 2025-05-08

**Authors:** Shilin Li, Taoyun Zhou, Yun Cheng, Xinyu Li

**Affiliations:** 1 School of Information, Hunan University of Humanities, Science and Technology, Loudi 417000, China; 2 College of Physics and Electronic Information Engineering & Key Laboratory of Low-Dimensional Structural Physics and Application, Guilin University of Technology, Guilin 541004, China

**Keywords:** aqueous zinc-ion batteries (AZIBs), in situ intercalation, polyaniline modification, electrochemical performance

## Abstract

With the rapid growth of renewable energy, the need for efficient and stable energy storage systems has become increasingly urgent. Aqueous zinc-ion batteries (AZIBs) can offer high safety, abundant zinc supply, and promising electrochemical properties. However, their performance is limited by poor electronic conductivity, slow Zn^2+^ diffusion, and structural degradation of conventional cathode materials. To address these issues, an in situ polyaniline (PANI) intercalation strategy for vanadium oxide cathodes is introduced in this paper. The conductive PANI chains play three key roles: (1) expand and stabilize interlayer spacing, (2) enhance electronic conductivity, and (3) provide mechanical support to prevent structural collapse and zinc-dendrite formation. A flower-like PANI-V_2_O_5_ hybrid is synthesized via synchronous oxidative polymerization, forming a hierarchical architecture without inert intercalants. The resulting electrode achieves a high specific capacity of 450 mAh·g^−1^ at 0.1 A·g^−1^ and retains 96.7% of its capacity after 300 cycles at 1 A·g^−1^, with excellent rate performance. These findings demonstrate that PANI intercalation enhances ion transport, electronic conductivity, and structural integrity, offering a promising design approach for next-generation AZIBs cathodes.

## 1. Introduction

In recent years, coal-based energy has caused serious environmental challenges, threatening sustainable development worldwide [1,2,3]. In response, renewable electricity sources have become key components of modern energy systems [4,5,6]. Among them, clean technologies such as wind and solar power play a vital role in energy conversion. However, their intermittent nature demands efficient and stable energy storage solutions, with secondary batteries—especially lithium-ion batteries (LIBs)—serving as the dominant technology [7,8].

Despite their wide application, LIBs face critical issues, including limited lithium resources, uneven global distribution, and safety risks posed by flammable organic electrolytes [9,10,11]. These drawbacks have motivated the search for alternative battery chemistries that are safer, more sustainable, and based on abundant elements.

Aqueous zinc-ion batteries (AZIBs) have attracted growing interest due to zinc’s natural abundance, low cost, favorable redox potential (−0.763 V vs. SHE), and reversible plating/stripping behavior in mildly acidic electrolytes (pH 3.6~6.0), which effectively suppress dendrite growth and side-product formation [12,13,14,15,16]. Moreover, the high density and two-electron transfer of Zn^2+^ provide AZIBs with a theoretical volumetric capacity (~5855 mAh·cm^−3^), surpassing that of LIBs (~2061 mAh·cm^−3^), making them ideal for compact and safe energy storage applications [17].

It is also important to note that in such aqueous electrolytes, both Zn^2^⁺ and H⁺ ions can participate in the electrochemical process. Proton insertion, particularly during the early stages of cycling, may contribute to capacity and influence redox kinetics—a phenomenon supported by recent studies [12].

Compared to LIBs, Zn^2+^ exhibits stronger electrostatic interactions with host materials, resulting in sluggish ion diffusion and capacity fading [18]. To address this, pre-intercalation strategies have been proposed to expand the interlayer spacing of vanadium-based materials and improve Zn^2+^ transport kinetics [19,20,21]. These strategies involve either ion intercalation or molecular intercalation. While the former can stabilize layered structures, the latter—especially using conductive polymers—has shown greater effectiveness. Owing to their larger molecular size, conductive polymers can significantly expand the interlayer spacing, facilitating ion transport while maintaining structural stability throughout cycling [22,23,24,25,26]. Furthermore, the insolubility of conductive polymers in electrolytes helps maintain the stability of the intercalated structure during cycling.

Among various vanadium-based oxides, V_2_O_5_ has been widely explored as a cathode material for AZIBs due to its multiple valence states (V^+2^ to V^+5^), high theoretical capacity (589 mAh·g^−1^), and layered crystal structure composed of VO_5_ square pyramids linked into sheets [27,28,29]. This structure provides ideal channels for Zn^2^⁺ intercalation and deintercalation. However, V_2_O_5_ suffers from poor electronic conductivity, slow ion diffusion, and structural degradation, limiting its long-term performance [29].

PANI, a conductive polymer, offers a promising solution. When intercalated into V_2_O_5_, it serves three synergistic roles: (1) acting as molecular spacers to enlarge and stabilize interlayer spacing; (2) providing continuous electron-conducting pathways; and (3) enhancing mechanical integrity to suppress structural collapse and vanadium dissolution during cycling.

Previous work by Chou et al. demonstrated that V_2_O_5_-PANI superlattices prevented vanadium leaching, increased interlayer distance, and weakened Zn^2^⁺-O^2−^ interactions, thereby improving Zn^2^⁺ reversibility [30]. Similarly, composites such as PEDOT@YVO have shown enhanced capacity and cycle life due to the presence of conductive polymers [31].

Building upon these insights, this paper proposes a molecular intercalation strategy via synchronous oxidative polymerization to in situ embed PANI into the V_2_O_5_ lattice—without the use of inert intercalants. The resulting flower-like organic–inorganic hybrid (PANI-V_2_O_5_) features tunable interlayer spacing and a hierarchical architecture. The intercalated PANI facilitates Zn^2+^ diffusion, improves electrical conductivity, and enhances structural stability.

Electrochemical evaluations confirm that the PANI-V_2_O_5_ composite achieves high specific capacity, excellent performance rate, and outstanding cycling stability. This paper presents a versatile design framework for the development of high-performance, durable AZIBs cathode materials.

## 2. Materials and Methods

### 2.1. Materials

The materials and equipment used in the experiments are listed in Table 1. To comprehensively investigate the structural, morphological, compositional, and electrochemical characteristics of the synthesized PANI-V_2_O_5_ composite, a series of characterization techniques are employed. Sample preparation is conducted following established protocols specific to each technique to ensure accuracy and reproducibility. Table 2 presents a summary of the instruments, operating parameters, measurement conditions, and software used for data acquisition and analysis.

### 2.2. Synthesis of PANI-V_2_O_5_

In this paper, a simple hydrothermal method is employed to synthesize a PANI-V_2_O_5_ organic–inorganic composite. The specific steps are as follows: Specifically, 1.5 g of ammonium metavanadate (NH_4_VO_3_) is dissolved in 60 mL of deionized water under ultrasonic agitation for 30 min to obtain a clear light-yellow solution. The mixture is then cooled to 0 °C, and 2 mL of aniline (C_6_H_7_N, AR grade) is added dropwise under continuous stirring. Meanwhile, the pH of the solution is adjusted to approximately 3 using 2.2 M dilute hydrochloric acid.

After stirring for 5 h, the resulting solution is transferred into a Teflon-lined stainless-steel autoclave and subjected to hydrothermal treatment at 140 °C for 24 h. Upon completion, the precipitated product is collected, thoroughly washed with deionized water and ethanol to remove residual impurities, and subsequently dried in an oven at 60 °C for 12 h. The final product obtained is the PANI-V_2_O_5_ composite material.

### 2.3. Electrode Preparation and Battery Assembly

(1) Reference and counter-electrodes: High-purity Zn foil (99.9%) is used as both the reference electrode (Zn/Zn^2+^ couple) and counter-electrode. Owing to its reversible redox behavior, Zn foil provides a stable potential of approximately 0.76 V vs. SHE in 3 M Zn(CF_3_SO_3_)_2_, ensuring reliable and reproducible electrochemical measurements.

(2) Working electrode: The active material (PANI-V_2_O_5_ or pristine V_2_O_5_), Super-P conductive carbon, and PVDF binder are mixed in a mass ratio of 7:2:1 and ground into a uniform slurry using N-methyl-2-pyrrolidone (NMP) as solvent. The slurry is then coated onto stainless-steel mesh current collectors and dried under vacuum at 65 °C for 12 h. The active mass loading is maintained at approximately 2 mg cm^−2^.

(3) Cell assembly: CR2016 coin cells are assembled in ambient conditions. The cell configuration included Zn foil as both reference and counter-electrodes, the prepared working electrode, a glass fiber separator (Whatman, Maidstone, UK), and 3 M Zn(CF_3_SO_3_)_2_ aqueous solution as the electrolyte.

(4) Electrochemical measurements: Galvanostatic charge/discharge (GCD) tests are conducted on a Neware CT 4008 battery testing system (Neware, Shenzhen, China) within a voltage window of 0.2–1.4 V (vs. Zn/Zn^2^⁺) at various current densities to assess rate capability and cycling stability.

Cyclic voltammetry (CV) and electrochemical impedance spectroscopy (EIS) are performed on a CHI760E electrochemical workstation (Shanghai Chenhua Instruments Corporation, Shanghai, China). CV tests are carried out at scan rates ranging from 0.1 to 1.0 mV·s^−1^ within the same voltage range. EIS measurements are performed after full charge, spanning a frequency range of 100 kHz to 0.01 Hz, with a 5 mV AC amplitude to evaluate interfacial resistance and ion transport kinetics. All electrochemical measurements are conducted at room temperature in a two-electrode configuration. Data acquisition and analysis are carried out using CHI software 14.01, Neware BTSDA 7.6, and OriginPro 2024 for curve fitting and graphical presentation.

## 3. **Results and Discussion**

### 3.1. Material Characterization

The structural, morphological, and chemical properties of the synthesized PANI-V_2_O_5_ composites are comprehensively characterized using a range of analytical techniques. Powdered samples are prepared according to the specific requirements of each method—for example, deposition on conductive substrates for XPS analysis, gold-sputter coating for SEM, and dispersion in ethanol for TEM grid preparation.

Surface morphology and elemental distribution are examined using a scanning electron microscope (Hitachi S-4800,Hitachi High-Technologies, Tokyo, Japan) equipped with an energy-dispersive X-ray spectrometer (EDS). High-resolution internal structural features are observed using transmission electron microscopy (JEOL JEM-2100,JEOL Ltd., Tokyo, Japan). The specific surface area (SSA) and pore size distribution are determined by nitrogen adsorption–desorption isotherms at −196 °C, using the Brunauer–Emmett–Teller (BET) and Barrett–Joyner–Halenda (BJH) methods (ASAP 2460 instrument, Micromeritics Instrument Corp., Norcross, GA, USA). The crystalline phases of the samples are identified via X-ray diffraction (Rigaku D/MAX-2500, Rigaku Corporation, Tokyo, Japan), while surface elemental compositions and oxidation states are analyzed by X-ray photoelectron spectroscopy (Thermo ESCALAB 250Xi, Thermo Fisher Scientific, Waltham, MA, USA).

Raman spectroscopy is performed using a Horiba LabRAM HR Evolution spectrometer with a 512 nm laser excitation source to probe molecular structure and bonding environments. Thermal stability and decomposition behavior are assessed by thermogravimetric/differential thermal analysis (SDT Q600,TA Instruments, New Castle, DE, USA), carried out from room temperature to 800 °C at a heating rate of 10 °C·min^−1^ in air.

#### 3.1.1. Scanning Electron Microscopy (SEM) and Energy Dispersive Spectroscopy (EDS)

**Figure 1:**
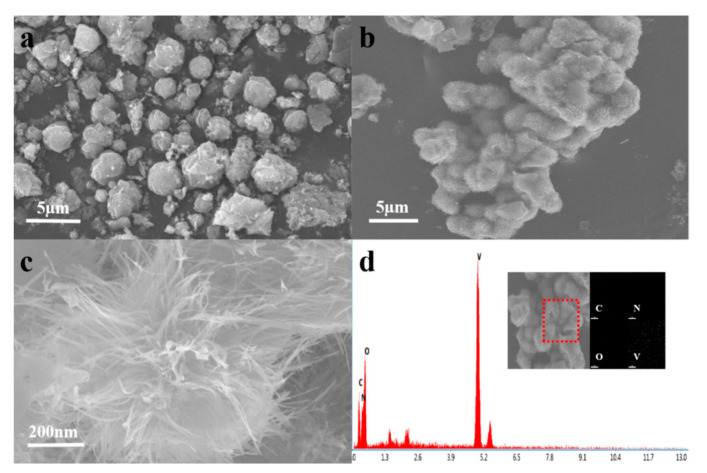
(**a**) SEM image of pure V_2_O_5_. (**b**,**c**) SEM images of PANI-V_2_O_5_, showing its microstructural changes. (**d**) EDS image of PANI-V_2_O_5_, illustrating the elemental composition and distribution.

The microstructure and elemental composition of the V_2_O_5_ and PANI-V_2_O_5_ composite are characterized using SEM and EDS, as shown in Figure 1.

Figure 1a displays the SEM image of pristine V_2_O_5_ synthesized without the addition of aniline. The material exhibits irregular spherical particles with rough surfaces, uniform size distribution, and a densely packed arrangement. This compact morphology may hinder ion transport and limit electrochemical performance.

In contrast, the morphology of PANI-V_2_O_5_ (Figure 1b) changes significantly upon PANI incorporation. The surface becomes more porous and less compact, with the appearance of sea-urchin-like protrusions ranging from approximately 2 to 5 nm. These structural changes suggest that PANI doping induces rearrangement of the V_2_O_5_ framework, leading to improved particle dispersion. The resulting porous architecture increases the surface area and enhances electrolyte ion accessibility, contributing to improved electrochemical behavior.

A magnified SEM image of PANI-V_2_O_5_ is shown in Figure 1c, revealing well-defined nanofiber-like structures with diameters in the tens of nanometers. These nanofibers are attributed to the growth of PANI, confirming its successful integration. The nanostructures provide abundant active sites and reduce ion diffusion distances, thereby enhancing charge transfer kinetics.

Figure 1d presents the EDS analysis, which confirms the presence of vanadium (V), oxygen (O), carbon (C), and nitrogen (N) in the composite. The energy spectrum of the left red curve is collected within the red box area. The uniform distribution of C and N elements—originating from PANI—further supports the successful intercalation of PANI into the V_2_O_5_ matrix. Elemental mapping (inset) shows homogeneous dispersion without signs of phase separation, indicating good structural stability. This uniformity plays a key role in maintaining electrochemical stability during cycling.

#### 3.1.2. Transmission Electron Microscopy (TEM) Analysis

**Figure 2:**
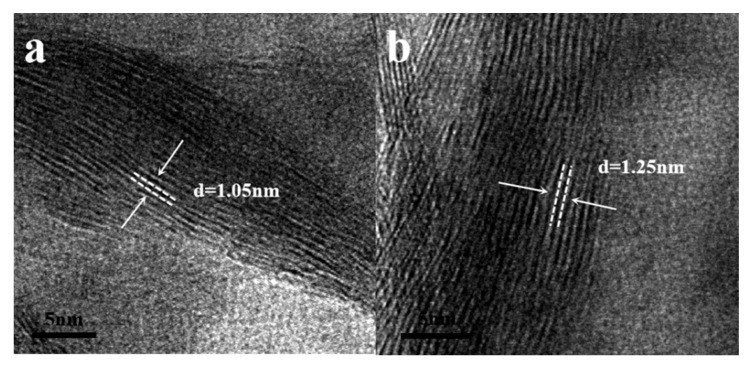
High-resolution TEM images: (**a**) V_2_O_5_ and (**b**) PANI-V_2_O_5._

To further elucidate the microstructural features of PANI-V_2_O_5_ composite, high-resolution TEM analysis is performed, as shown in Figure 2.

Figure 2a presents the TEM image of pristine V_2_O_5_, revealing a well-ordered, tightly stacked layered structure with an interlayer spacing of approximately 1.05 nm. In contrast, Figure 2b shows the morphology of the PANI-V_2_O_5_ composite. Upon PANI intercalation, the interlayer spacing expands to approximately 1.25 nm, indicating a more open and less compact layered structure.

The increase in interlayer distance is attributed to the successful intercalation of PANI chains, which introduce additional electrostatic repulsion and steric hindrance between adjacent V_2_O_5_ layers. The expanded spacing facilitates the diffusion of electrolyte ions, reduces charge transfer resistance, and enhances ion transport kinetics.

Overall, the PANI-induced structural modification not only alters the lamellar architecture of V_2_O_5_ but also improves its electrochemical properties, offering enhanced capacity and cycling stability. These results highlight the potential of PANI-V_2_O_5_ as an efficient cathode material for high-performance energy storage devices.

#### 3.1.3. X-Ray Diffraction (XRD) and Raman Spectroscopy Analysis

**Figure 3:**
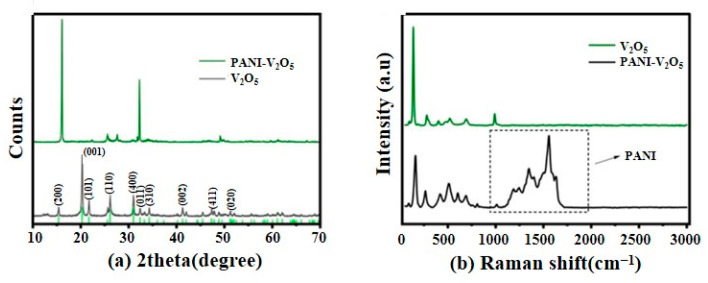
Characterization of pure V_2_O_5_ and PANI-V_2_O_5_ composite materials: (**a**) X-ray diffraction (XRD) patterns and (**b**) Raman spectroscopy comparative analysis.

The phase structure and molecular interactions of the synthesized materials are characterized using XRD and Raman spectroscopy, as presented in Figure 3.

Figure 3a shows the XRD patterns of pristine V_2_O_5_ (black curve) and the PANI-V_2_O_5_ composite (green curve). The V_2_O_5_ sample displays distinct diffraction peaks corresponding to the (001), (200), and (300) crystal planes, confirming its typical layered structure. In the PANI-V_2_O_5_ spectrum, these characteristic peaks are retained, indicating that the layered framework remains intact after polyaniline intercalation. Notably, the (001) peak shifts toward a lower angle, suggesting an expansion of interlayer spacing due to the insertion of PANI chains. Crucially, no additional peaks associated with other vanadium oxide phases such as VO_2_ or V_2_O_3_ are detected, confirming that the structural modification originates exclusively from PANI incorporation. These results align well with TEM observations, supporting the successful and selective intercalation of PANI.

To further probe molecular interactions and bonding environments, Raman spectroscopy is performed in the 0–3000 cm^−1^ range, as shown in Figure 3b. The Raman spectrum of pure V_2_O_5_ (green curve) exhibits characteristic V-O vibrational bands. In the PANI-V_2_O_5_ composite (black curve), these peaks are preserved, while additional bands emerge at 1366, 1453, 1532, and 1562 cm^−1^. These are assigned to C-C stretching and symmetric/asymmetric C=C vibrations from the polyaniline backbone. No Raman signals corresponding to VO_2_ or V_2_O_3_ are observed, confirming the phase purity of the composite. The inset in Figure 3b highlights the unique PANI-related vibrational features, further corroborating its successful incorporation and possible formation of a supramolecular framework within the V_2_O_5_ layers.

#### 3.1.4. X-Ray Photoelectron Spectroscopy (XPS) Analysis

**Figure 4:**
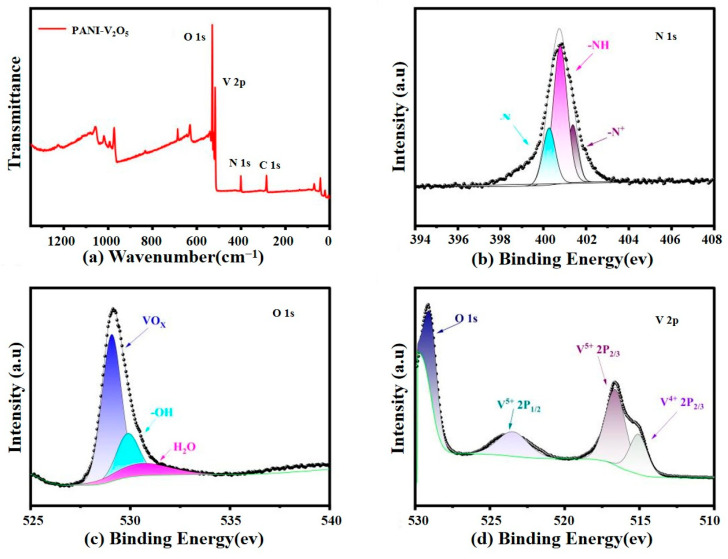
XPS analysis results: (**a**) full XPS spectrum of the PANI-V_2_O_5_ sample, (**b**) high-resolution N 1s spectrum of PANI-V_2_O_5_, (**c**) high-resolution O 1s spectrum of PANI-V_2_O_5_, and (**d**) high-resolution V 2p spectrum of PANI-V_2_O_5_.

XPS is employed to analyze the surface chemical composition and valence states of the elements in the PANI-V_2_O_5_ composite. The results, including the survey and high-resolution spectra, are shown in Figure 4.

Figure 4a displays the full XPS spectrum, which confirms the presence of C 1s, N 1s, O 1s, and V 2p signals. The prominent O and V peaks verify that vanadium pentoxide remains the dominant component of the material. The clear detection of C and N peaks, originating from the polyaniline backbone, confirms the successful incorporation of PANI within the composite structure.

The high-resolution N 1s spectrum (Figure 4b) is deconvoluted into three peaks: ~399 eV (N^−^, attributed to the aniline group), ~400 eV (-NH-, corresponding to the imine unit in the PANI), and ~401 eV (N^+^, associated with doped or protonated structures). These correspond to quinonoid (400.38 eV), benzenoid (400.73 eV), and quaternary ammonium (401.23 eV) species, respectively. The coexistence of multiple nitrogen species indicates various oxidation states of PANI, which can enhance electronic conductivity and support redox activity during battery operation.

Figure 4c shows the O 1s spectrum, which reveals three distinct components: ~530.3 eV (VO_X_ species from the V_2_O_5_ lattice), 531.8 eV (-OH groups), and 533.5 eV (H_2_O adsorption). The presence of hydroxyl and water species suggests surface interactions due to PANI doping and/or environmental exposure. These oxygen species may contribute to modified electronic structure and improved ion transport.

The high-resolution V 2p spectrum (Figure 4d) reveals the coexistence of V^4^⁺ and V^5^⁺ states. Peaks at 516.08 eV (V 2p_3_/_2_) and 523.78 eV (V 2p_1_/_2_) correspond to V^4^⁺, while the peaks at 517.28 eV and 524.98 eV are assigned to V^5^⁺. This mixed valence state indicates partial surface reduction of V_2_O_5_, possibly forming VO_2_-like species. However, no distinct VO_2_ phase is detected by XRD or Raman spectroscopy, suggesting that the reduced vanadium species are limited to the surface and do not constitute a separate bulk phase.

#### 3.1.5. Nitrogen Adsorption–Desorption Analysis

**Figure 5:**
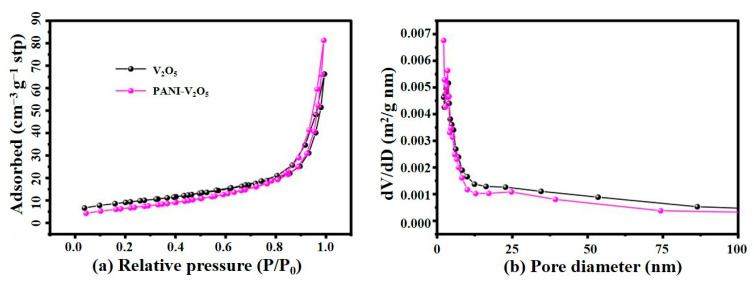
(**a**) Nitrogen adsorption–desorption isotherms of PANI-V_2_O_5_ and V_2_O_5_. (**b**) Corresponding pore size distribution curves.

To further examine the porosity and surface characteristics of the synthesized materials, nitrogen adsorption–desorption measurements are carried out on both the PANI-V_2_O_5_ and pristine V_2_O_5_ composite. The results are presented in Figure 5.

Figure 5a shows the nitrogen adsorption–desorption isotherms of the two samples. Both materials exhibit type IV isotherms with H3-type hysteresis loops, characteristic of mesoporous structures. At low relative pressures (P/P_0_ < 0.2), the adsorption volume remains low, indicating a negligible contribution from micropores. As P/P_0_ increases above 0.8, a notable increase in adsorption is observed, particularly for the PANI-V_2_O_5_ sample.

The specific surface area of V_2_O_5_ is calculated to be 66.13 m^2^·g^−1^, while that of PANI-V_2_O_5_ increases to 81.28 m^2^·g^−1^. This enhancement can be attributed to the incorporation of polyaniline, which increases the accessible porosity, introduces additional surface-active sites, and improves electrolyte wettability—all beneficial for Zn^2+^ storage and electrochemical performance.

Figure 5b presents the pore size distribution profiles of the materials, derived from the desorption branch using the Barrett–Joyner–Halenda (BJH) method. The desorption branch is selected due to its higher reliability in assessing mesoporous characteristics, especially in the capillary condensation region. Both samples exhibit dominant pore diameters below 25 nm, confirming their mesoporous nature.

Notably, the PANI-V_2_O_5_ composite displays a broader pore size distribution with pronounced peaks at approximately 2.21 nm and 17.19 nm. This indicates that PANI doping not only increases total pore volume but also creates a hierarchical pore network. Such architecture facilitates rapid Zn^2^⁺ diffusion, enhances electrolyte penetration, and provides efficient electron pathways, contributing to the improved rate performance and long-term cycling stability of the electrode material.

### 3.2. Characterization by Electrochemistry

To evaluate the Zn^2^⁺ storage capability of the synthesized material as a cathode for AZIBs, CR2016-type coin cells are assembled under ambient conditions, as detailed in the Materials and Methods section.

#### 3.2.1. Cyclic Voltammetry (CV) Tests

CV is conducted using a CHI-760E electrochemical workstation to assess the redox behavior and reversibility of the PANI-V_2_O_5_ electrode. The tests are performed at room temperature using a two-electrode configuration, with high-purity Zn foil serving as both the reference and counter-electrode. The working electrode is fabricated by coating the synthesized material onto a stainless-steel mesh. A 3 M Zn(CF_3_SO_3_)_2_ aqueous solution served as the electrolyte, and a glass fiber membrane is used as the separator.

**Figure 6:**
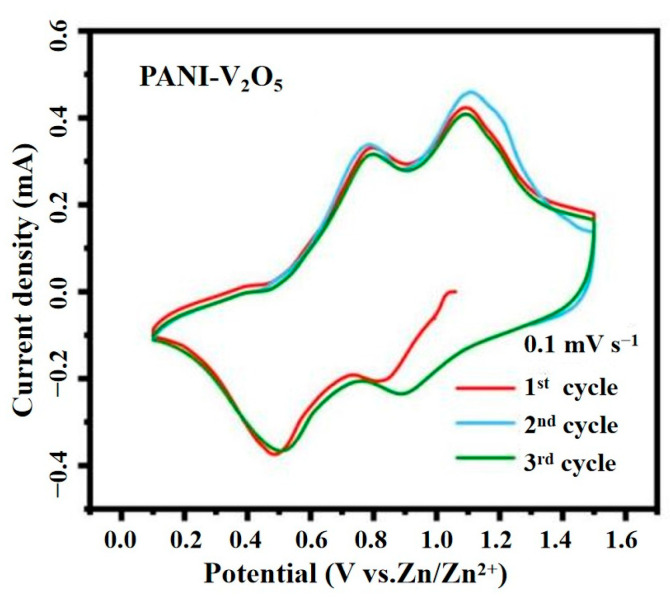
CV curves of PANI-V_2_O_5_ at a scan rate of 0.1 mV·s^−1^.

CV measurements are recorded within a voltage window of 0.2 to 1.4 V (vs. Zn/Zn^2^⁺) at scan rates ranging from 0.1 to 1.0 mV·s^−1^. Figure 6 displays the CV curves of the PANI-V_2_O_5_ electrode at 0.1 mV·s^−1^ for the first three cycles.

Two distinct cathodic peaks, centered at 0.45 V and 0.90 V, are observed, corresponding to the Zn^2^⁺ intercalation into the electrode matrix. Conversely, anodic peaks at approximately 0.80 V and 1.10 V reflect the Zn^2^⁺ deintercalation process. These well-defined redox peaks confirm that the electrode operates via a reversible Zn^2^⁺ intercalation/deintercalation mechanism.

The high degree of overlap among the second and third CV cycles indicates excellent electrochemical reversibility and stable reaction kinetics. The slight deviation observed in the first cycle is attributed to the initial activation of surface sites, after which the electrode structure stabilizes, leading to consistent redox behavior in subsequent scans.

The incorporation of PANI is instrumental in enhancing the electrode’s electrochemical response. First, PANI’s intrinsic electrical conductivity facilitates efficient electron transport, thereby accelerating charge transfer during cycling. Second, its mesoporous architecture improves electrolyte accessibility and ion diffusion, thus supporting rapid Zn^2^⁺ transport. This synergistic enhancement leads to improved specific capacity and cycling stability, highlighting the efficacy of the PANI intercalation strategy.

#### 3.2.2. Galvanostatic Charge–Discharge (GCD) Tests

GCD tests are conducted to evaluate the rate capability and long-term cycling stability of the synthesized PANI-V_2_O_5_ composite. Measurements are performed using a Neware CT 4008 battery testing system at room temperature. The assembled CR2016 coin cells consisted of a working electrode fabricated by coating a slurry of the active material (PANI–V_2_O_5_ or V_2_O_5_),Super P conductive carbon, and PVDF binder (mass ratio 7:2:1) onto a stainless steel mesh current collector. The mass loading of the active material is approximately 2 mg cm^−2^. High-purity Zn foil is employed as both the counter and reference electrodes, 3 M Zn(CF_3_SO_3_)_2_ serves as the electrolyte, and a glass fiber membrane is used as the separator.

**Figure 7:**
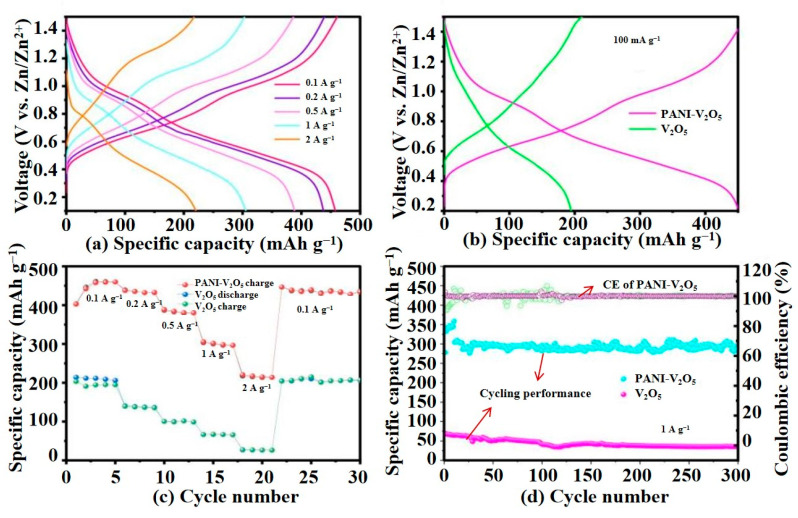
Comparison of electrochemical performance between PANI-V_2_O_5_ and V_2_O_5_. (**a**) First charge–discharge curves of PANI-V_2_O_5_ at different current densities. (**b**) Comparison of charge–discharge curves of PANI-V_2_O_5_ and V_2_O_5_ at 0.1 A g^−1^. (**c**) Rate performance of the two materials at different current densities. (**d**) Cycling stability and coulombic efficiency trends of PANI-V_2_O_5_ and V_2_O_5_ at 1 A g^−1^ current density, the green curve represents the specific capacity cycling performance of PANI-V_2_O_5_.

Figure 7a presents the charge–discharge profiles of PANI-V_2_O_5_ electrode at various current densities (0.1, 0.2, 0.5, 1, and 2 A·g^−1^). At 0.1 A·g^−1^, the electrode delivers a high specific capacity of 450 mAh·g^−1^. As the current density increases, the capacity gradually decreases due to kinetic limitations but remains relatively high, indicating good rate capability and structural resilience under fast charge–discharge conditions.

Figure 7b compares the charge–discharge curves of PANI-V_2_O_5_ and the pristine V_2_O_5_ at a current density of 0.1 A·g^−1^. The PANI-V_2_O_5_ composite achieves a much higher discharge capacity (450 mAh·g^−1^) compared to V_2_O_5_ (194 mAh·g^−1^). This enhancement is attributed to the PANI intercalation, which increases electrical conductivity, expands the interlayer spacing, and improves ion diffusion kinetics.

The rate performance results in Figure 7c further highlight the superiority of the PANI-V_2_O_5_ electrode. The specific capacities at 0.1, 0.2, 0.5, 1, and 2 A·g^−1^ are 450, 435, 384, 305, and 220 mAh·g^−1^, respectively. Notably, when the current density is reverted to 0.1 A·g^−1^, the capacity is fully recovered to 450 mAh·g^−1^, indicating excellent reversibility and structural integrity during high-rate cycling. In contrast, the pristine V_2_O_5_ electrode exhibits more pronounced capacity degradation at higher rates, suggesting inferior ion transport kinetics and structural stability.

Figure 7d shows the long-term cycling performance at a current density of 1 A·g^−1^. While the capacity of V_2_O_5_ declines sharply—retaining only 51.1% after 300 cycles—the PANI-V_2_O_5_ electrode maintains 96.7% of its initial capacity with a low average capacity decay rate of 0.0233% per cycle. The coulombic efficiency remains close to 100% throughout the test, reflecting excellent electrochemical reversibility and minimal side reactions.

These results demonstrate that the integration of polyaniline significantly improves the performance rate and cycling durability of V_2_O_5_, validating the effectiveness of the molecular intercalation strategy for high-performance aqueous zinc-ion batteries.

#### 3.2.3. Electrochemical Kinetics Analysis

**Figure 8:**
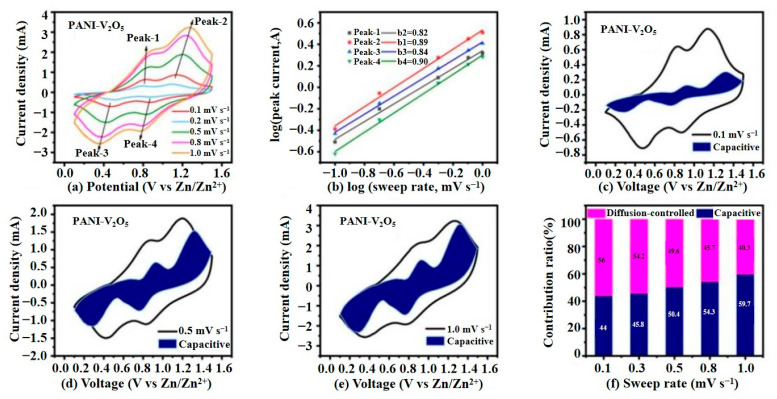
Electrochemical kinetic characteristics of the PANI-V_2_O_5_ electrode. (**a**) Cyclic voltammetry (CV) curves of PANI-V_2_O_5_ at different scan rates. (**b**) Log(i) vs. log(v) relationship curve corresponding to the oxidation and reduction peaks in the CV. (**c**–**e**) Pseudocapacitive contribution separation curves at scan rates of 0.1, 0.5, and 1.0 mV s^−1^. (**f**) Comparison of capacitive storage and diffusion-controlled contributions at different scan rates.

To gain deeper insight into the charge storage mechanism of the PANI-V_2_O_5_ electrode, CV tests are conducted at various scan rates, ranging from 0.1 to 1.0 mV·s^−1^. Figure 8 presents a comprehensive kinetic analysis, focusing on redox behavior, pseudocapacitive contribution, and scan rate dependence.

As shown in Figure 8a, the CV curves of PANI-V_2_O_5_ retain their shape across all scan rates, suggesting stable and reversible electrochemical processes. With increasing scan rates, both anodic and cathodic peaks shift oxidation peaks toward higher and reduction peaks toward lower potentials, indicating increased polarization due to kinetic limitations. Despite these shifts, the preservation of peak shapes confirms the electrode’s robust reaction reversibility.

To analyze the charge storage mechanism, the relationship between peak current (*i*) and scan rate (*v*) is examined using the power-law equation:

(1) $$i=av^{b}$$

where *a* and *b* are constants. A *b* value near 0.5 indicates a diffusion-controlled process, while values approaching 1.0 suggest surface-controlled pseudocapacitive behavior.

As shown in Figure 8b, the log(*i*)–log(*v*) plots yield *b* values of 0.82, 0.89, 0.84, and 0.90 for the four redox peaks, respectively. These results indicate that charge storage in PANI-V_2_O_5_ is primarily governed by pseudocapacitive processes rather than slow ion diffusion.

To quantify the pseudocapacitive contribution, the current response is further deconvoluted using the following equation [32]:

(2) $$i=k_{1}v+k_{2}v^{0.5}$$

where $k_{1}v$ represents the capacitive (surface-controlled) current, and $k_{2}v^{0.5}$ denotes the diffusion-controlled current.

Figure 8c–e show this separation at scan rates of 0.1, 0.5, and 1.0 mV·s^−1^. At 0.1 mV·s^−1^, the pseudocapacitive contribution is approximately 44%. This value increases with the scan rate, reaching 59.7% at 1.0 mV·s^−1^, illustrating the dominant role of pseudocapacitance at high rates.

Figure 8f summarizes the pseudocapacitive and diffusion-controlled contributions across all tested scan rates. At low scan rates, diffusion processes dominate, facilitating Zn^2^⁺ insertion/extraction into/from the bulk material. As the scan rate increases, the pseudocapacitive contribution grows, enabling fast charge–discharge behavior and enhancing rate capability. This high pseudocapacitive contribution is a direct result of PANI intercalation, which provides a highly conductive, porous matrix that supports rapid surface redox reactions and effective Zn^2^⁺ transport. These properties underpin the outstanding rate capability and cycling stability observed in previous tests.

To benchmark the performance of the PANI-V_2_O_5_ cathode, a comparison with recent State-of-the-Art AZIB systems is summarized in Table 3 [33,34]. While those studies demonstrate impressive ultra-long-term stability or novel electrolyte strategies, this work presents a highly competitive balance of capacity, rate capability, and practical stability.

While other systems report ultra-long cycle lives or advanced electrolyte formulations, the PANI-V_2_O_5_ composite demonstrates a balanced performance profile, achieving high capacity (450 mAh·g^−1^), excellent rate capability, and stable cycling (96.7% retention after 300 cycles at 1 A·g^−1^). Importantly, this is achieved using a practical, low-cost Zn(CF_3_SO_3_)_2_ electrolyte; avoiding expensive or complex chemistries; and offering scalability for real-world applications.

Furthermore, the unique advantage of this paper lies in the in situ intercalation of PANI, which not only enhances the electronic conductivity and buffers structural deformation during cycling but also actively contributes to the charge storage through its intrinsic surface redox activity. This dual-functionality—combining the intercalation capabilities of layered V_2_O_5_ with the pseudocapacitive behavior of conductive PANI—sets this system apart from conventional carbon-based or metal-oxide cathodes. The result is a well-balanced electrode material that delivers both high capacity and robust cycling stability.

In conclusion, the PANI-V_2_O_5_ hybrid electrode developed in this paper offers a synergistic mechanism that integrates high specific capacity, superior rate capability, and prolonged cycling performance under practical conditions. These attributes make it a compelling candidate for next-generation AZIBs, particularly for scalable, safe, and high-power energy storage applications.

## 4. Conclusions

In this paper, we report the in situ intercalation of PANI into V_2_O_5_ via synchronous oxidative polymerization. The resulting PANI-V_2_O_5_hybrid exhibits a flower-like structure and significantly improves electrochemical performance in AZIBs. This enhancement arises from the synergistic effect between V_2_O_5_ and PANI, forming a dual-function electrode that addresses the limitations of traditional oxide cathodes.

PANI improves performance in three main ways: (1) It acts as a nanoscopic spacer, expanding and stabilizing the V_2_O_5_ interlayer spacing. This facilitates fast, reversible Zn^2^⁺ intercalation and prevents structural collapse. (2) Its conductive framework enhances electron transport, improving rate capability. (3) The polymer matrix buffers volume changes and suppresses vanadium dissolution and dendrite formation, ensuring better cycling stability. As a result, the PANI-V_2_O_5_ electrode delivers a high specific capacity of 450 mAh g^−1^ at 0.1 A g^−1^ and retains 96.7% capacity after 300 cycles at 1 A g^−1^.

The molecular modification strategy offers a clear path for developing advanced AZIBs cathodes. Future work may optimize PANI by adjusting its molecular weight, adjusting its doping level, or introducing functional copolymers. Combining PANI-V_2_O_5_ with materials like graphene or MXene may further improve conductivity and ion transport. In situ and operando techniques are also recommended to better understand ion diffusion and structural evolution during cycling.

## Figures and Tables

**Table 1:** Experimental materials.

Material	Purity/Model	Manufacturer
Hydrochloric acid	AR	Xilong Chemical Corporation (Shantou, China)
Ammonium metavanadate	AR	Luoen Corporation (Shanghai, China)
Aniline	AR	Xilong Chemical Corporation (Shantou, China)
Hydrogen peroxide	30%AR	Xilong Chemical Corporation (Shantou, China)
Ethanol	AR	Xilong Chemical Corporation (Shantou, China)
Deionized water	AR	Laboratory preparation (Local Laboratory, China)
Fiberglass diaphragm	1823-047	Whatman (Maidstone, UK)
Ar	AR	Guangzhou Junduo Gas Corporation (Guangzhou, China)

**Table 2:** Summary of characterization techniques, instruments, and operating conditions.

Technique	Instrument	Manufacturer	Parameters	Conditions	Software
XRD	Rigaku D/MAX-2500	Rigaku Corporation, Tokyo, Japan	Cu Kα (λ = 1.5406 Å); 40 kV, 30 mA; 5°/min; 0.02° step	2θ range: 10~70°	MDI Jade 6.5
SEM & EDS	Hitachi S-4800	Hitachi High-Technologies, Tokyo, Japan	Accelerating voltage: 5~20 kV	vacuum mode	Quartz PCI 8.0, Oxford INCA 5.05
TEM	JEOL JEM-2100	JEOL Ltd., Tokyo, Japan	Accelerating voltage: 200 kV	vacuum mode	Digital Micrograph 3.30.2004.0
XPS	Thermo ESCALAB 250Xi	Thermo Fisher Scientific, Waltham, MA, USA	Al Kα (1486.6 eV); pass energy 20 eV; resolution 0.5 eV	Base pressure < 5 × 10^−9^ mbar	Thermo Avantage 5.991
Raman Spectroscopy	Horiba LabRAM HR Evolution	HORIBA Scientific, Kyoto, Japan	Excitation wavelength: 512 nm	Ambient temperature	LabSpec 6.5
BET/BJH	ASAP 2460	Micromeritics Instrument Corp., Norcross, GA, USA	Measurement at −196 °C	N_2_ isotherms for SSA	MicroActive 6.07
TGA/DTA	SDT Q600	TA Instruments, New Castle, DE, USA	Heating rate: 10 °C/min	Temp. range: RT to 800 °C in air	Universal Analysis 5.5
GCD	Neware CT4008	Neware, Shenzhen, China	0.2~1.4 V	Room temperature	Neware BTSDA 7.6
CV, EIS	CHI760E	Shanghai Chenhua Instruments Corporation, Shanghai, China	CV: 0.1~1.0 mV/s; EIS: 0.01 Hz~100 kHz	Room temperature	CHI Software 14.01

**Table 3:** Comparative analysis of long-term cycling performance in recent AZIB cathode materials.

Ref.	Cathode Material	Electrolyte	Current Density	Cycle Number	Capacity Retention (%)
This paper	PANI-V_2_O_5_	3 M Zn (CF_3_SO_3_)_2_	1 A g^−1^	300	96.7%
[33]	Poly(catechol)	4 M Zn (TFSI)_2_	30 C (~15 A g^−1^)	48,000	~83% (0.00035%fade/cycle)
[34]	Water-in-polymer AZIB (non-metal)	Polymer–salt gel	0.1 A g^−1^	8000	~80%

## Data Availability

All the datasets used in this manuscript are publicly available datasets already in the public domain.
